# Left ventricular pressure‐loading improves pressure‐induced right ventricular remodeling by redistributing mechanical load and reducing mechanosignaling

**DOI:** 10.14814/phy2.70546

**Published:** 2025-09-19

**Authors:** Xavier Alexander Lee, Sonja Raschzok, Jean‐Francois Desjardins, Tim Van Loon, Andrija Plavetic, Omar Kanny, Golam Kabir, Linda Nghiem, John Dauz, Andras Kapus, Joost Lumens, Kim A. Connelly, Mark K. Friedberg

**Affiliations:** ^1^ Keenan Research Centre for Biomedical Science, St. Michael's Hospital Toronto Ontario Canada; ^2^ Department of Physiology University of Toronto Toronto Ontario Canada; ^3^ Labatt Family Heart Centre, The Hospital for Sick Children Toronto Ontario Canada; ^4^ Biomedische Technologie School for Cardiovascular Diseases, Maastricht University Maastricht Netherlands

**Keywords:** cardiac remodeling, fibrosis, pressure‐overload, pulmonary artery banding, right ventricle

## Abstract

Right ventricular (RV) function under pressure overload (PO) is critical in congenital heart disease outcomes. While moderate left ventricular (LV) pressure‐loading has been shown to benefit RV function, the mechanisms and optimal degree of loading remain unclear. This study investigated whether increasing LV afterload could enhance RV function, remodeling, and molecular signaling. Using computational modeling and an in vivo “double‐banding” (DB) approach in Sprague–Dawley rats—constricting both the pulmonary artery (PA) and transverse aorta—the effects of LV loading were assessed. Modeling suggested that LV pressure‐loading improves RV contractility by homogenizing RV load. In vivo, DB rats exhibited higher tricuspid annular plane systolic excursion (TAPSE) compared to those with only pulmonary artery banding (PAB). Hemodynamic analysis showed reduced end‐diastolic pressure (EDP) and increased end‐diastolic pressure–volume relationship (EDPVR) in DB rats. Histological examination revealed less RV fibrosis in DB rats with moderate LV loading (DBmod) than in those with mild loading (DBmild) or PAB. Molecular studies indicated that markers of fibrosis and maladaptive signaling were elevated in PAB RVs but normalized or downregulated in DBmod RVs. These findings suggest that moderate LV pressure‐loading during RV‐PO improves RV remodeling and function, providing mechanistic insights and potential therapeutic strategies for congenital heart disease.

## 1 INTRODUCTION

In children and adults with various types of congenital heart disease (CHD) RV function in response to adverse loading is an important determinant of morbidity and mortality (Cho & Ma, 2013; Friedberg & Reddy, 2019; Guimaron et al., 2018). In response to chronic pressure overload, the RV progressively undergoes remodeling, which includes but is not limited to, a reduction in RV ejection fraction, contractility, and cardiac index, septal bowing towards the left ventricle (LV), inflammation, cardiomyocyte hypertrophy, and fibrosis (Avazmohammadi et al., 2017; Mendiola et al., 2023).

Confined within a common pericardium and sharing the septum and common myocardial fibers, the structure and function of one ventricle impacts the contralateral ventricle (Feneley et al., 1989; Henderson & Prince, 1914; Karunanithi et al., 2001; Naeije & Badagliacca, 2017). Despite their differing mechanical loads, much remains unknown about how each ventricle responds to pathology in the other. Animal models have been developed to model cardiac pressure‐loading states such as transverse aortic constriction (TAC) and pulmonary artery banding (PAB) (Dealmeida et al., 2010; Urashima et al., 2008). Although both models have been used to study pathological remodeling in their respective ventricles—relatively few studies (Belenkie et al., 1995; Kapur et al., 2013; Yamashita et al., 1989) have examined the impact of these interventions on the contralateral ventricle. We previously studied a rabbit model that underwent both PAB to induce severe RV hypertension and concomitant moderate aortic constriction, comparing them to rabbits that only underwent PAB (Apitz, Honjo, Humpl, et al., 2012). We found that adding LV pressure overload improved RV systolic function and reduced myocyte hypertrophy and RV collagen deposition in both ventricles, without impairing LV function. These results suggested that increased LV afterload enhances RV contractility and limits maladaptive RV remodeling during RV pressure‐loading. However, the mechanical basis for these benefits, the role of LV afterload magnitude, and the involvement of mechano‐sensitive molecular signaling in reversing RV fibrosis remain unknown.

Yes‐associated Protein (YAP) and transcriptional coactivator with PDZ‐binding motif (TAZ) are conserved molecular mechanotransducers that respond to a diverse array of mechanical cues such as extracellular matrix (ECM) rigidity, shear stress, and cell shape and translate these changes into cell‐specific transcriptional outputs (Panciera et al., 2017). We previously showed that TAZ and myocardin‐related transcription factor‐A (MRTF‐A) interact in the kidney to modulate pressure‐induced kidney fibrosis, and that this interaction was conserved in the RV following pressure overload (Bialik et al., 2019; Rzepka et al., 2025; Speight et al., 2016). Importantly, we demonstrated that MRTF‐A inhibition during PAB decreased RV fibrosis and slightly improved RV systolic function; these changes were accompanied by a decrease in fibroblast‐to‐myofibroblast transition which was dependent on a Ras homolog family member A (RhoA)/MRTF‐A/TAZ signaling axis (Rzepka et al., 2025).

Building on our previous work (Apitz, Honjo, Humpl, et al., 2012; Rzepka et al., 2025), we hypothesized that the reduction in PAB‐induced RV remodeling was dependent on the degree of aortic banding and correlated with downregulation of MRTF and TAZ mechanosensitive signaling. To test this, we compared the hemodynamic, mechanical, and molecular effects of simultaneous PAB and TAC versus PAB alone. Acute effects were assessed using a biophysical model, CircAdapt (Lumens et al., 2019), while long‐term functional and molecular changes were assessed in rats. Herein, we demonstrated that RV chamber shape and function improve when the LV is pressure‐overloaded by reducing diastolic mechanical discoordination between the RV and LV through the interventricular septum, that this response is dependent upon the degree of LV pressure‐loading and is associated with downregulation of TAZ‐MRTF molecular signaling.

## 2 METHODS

### 2.1 Biophysical model

We used the CircAdapt biophysical model (Lumens et al., 2009; Walmsley et al., 2015) to simulate cardiovascular mechanics and hemodynamics under healthy circumstances, during PAB, and during double banding with varying severities of aortic banding. CircAdapt is a computational model of the human cardiovascular system (www.CircAdapt.org) that allows for fast beat‐to‐beat simulation of cardiac and vascular mechanics and hemodynamics in both healthy and diseased conditions. The model consists of a network of different modules representing the main elements of the closed‐loop cardiovascular system, including atrial and ventricular cavities, myocardial tissues, cardiac valves, pericardium, large blood vessels, and peripheral systemic and pulmonary microvasculature.

In CircAdapt, ventricular mechanical interaction is simulated through the TriSeg module (Lumens et al., 2009). The ventricular geometry consists of three thick spherical segments representing the RV free‐wall (RVfw), LV free‐wall (LVfw), and the interventricular septum. These ventricular walls meet at a common junction forming the RV and LV cavities. The local myofiber stress–strain relation in each wall is governed by the three‐element Hill model, which describes active‐ and passive cardiac myofiber mechanics (Arts et al., 2003), including the well‐established myofiber force–velocity and force–length relationships. The septal geometry is determined based on the balance of tensile force acting on the junction as generated by each of the three ventricular walls. Previous studies have shown that this TriSeg module realistically simulates cardiac mechanics, hemodynamics, and septal deformation in cases of RV failure and pulmonary arterial hypertension (PAH) (Palau‐Caballero et al., 2017).

### 2.2 Computer simulation protocol

**FIGURE 1:**
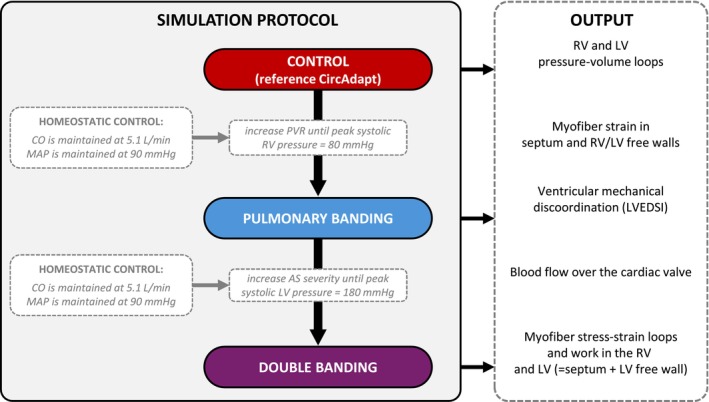
Overview of computational methodologies. Simple overview of the CircAdapt simulation protocol including input parameters and outputs of interests. The pulmonary banding simulation was achieved by increasing PVR such that the resulting RV systolic peak pressure was 80 mmHg. The double band simulation was achieved by decreasing the aortic valve orifice area such that the resulting peak LV systolic pressure was 180 mmHg. To mimic homeostatic regulation, the pressure‐flow control module was utilized to maintain a CO of 5.1 L/min and a mean systemic arterial pressure of 92 mmHg. CO, cardiac output; LV, left ventricle; PVR, pulmonary vascular resistance; RV, right ventricle.

A schematic overview of the simulation protocol and output is shown in Figure 1. In brief, the PAB model was created by increasing the pulmonary vascular resistance (PVR) from 2.0WU[dyn·s/cm^5^] (CONTROL) to 11.0WU, resulting in an RV systolic peak pressure of 80 mmHg, which represents a severe PAH or RV pressure‐loading substrate (Stout et al., 2019). To mimic homeostatic regulation, the pressure‐flow control module was utilized to maintain a cardiac output of 5.1 L/min and a mean systemic arterial pressure of 92 mmHg, while heart rate was kept constant. This module adjusts stressed blood volume and systemic vascular resistance in response to hemodynamic changes, thereby stabilizing flow and pressure without invoking explicit autonomic reflex mechanisms. No additional adaptation or remodeling mechanisms (e.g., changes in ventricular geometry, wall mass, or passive stiffness) were included. This assumption allows investigating the acute mechanical and hemodynamic effects of pulmonary and double banding.

Next, double banding was simulated by decreasing the aortic valve orifice area from 5.00cm^2^ (CONTROL and PAB) to 0.50cm^2^, in steps of 0.20cm^2^. The gradual decrease in aortic valve orifice area allows us to study the acute effect of different degrees of aortic banding on RV function. Similar to the PAB simulation, cardiac output, mean systemic arterial pressure, and heart rate were maintained, and no cardiac remodeling was performed.

### 2.3 In silico assessment of cardiac function in pulmonary and double banding

To gain a mechanistic understanding of the potential factors underlying RV remodeling in PAB and double banding, various indices of RV tissue and pump function were quantified in each simulation. End‐systolic elastance (E_es_) was calculated to assess RV contractile function as defined by the slope of the RV pressure–volume relation at end‐systole after preload manipulation (Maughan et al., 1979).

To measure the degree of ventricular mechanical discoordination between the LV and RV, we calculated the LV early diastolic strain index (LVEDSI). LVEDSI is defined as the ratio between the difference in LV strain during the time span between LVfw and RVfw peak strain and the total LVfw peak strain. Larger values suggest greater discoordination. In a previous publication (Lumens et al., 2012), LVEDSI was proposed as an indicator for poor RV function in pulmonary hypertension and associated with inadequate RV structural adaptation and increased RVfw myofiber load relative to LV myofiber load.

Furthermore, local myofiber work in the RVfw, LVfw, and interventricular septum was quantified to gain insights into ventricular tissue and pump efficiency, which may have implications for long‐term remodeling (Ryo et al., 2015). Work density was calculated from the area of the local myofiber stress–strain loop corrected for wall mass.

Lastly, mean right‐ (RA) and left atrial (LA) pressures were calculated to provide insights into the hemodynamic consequences within the heart chambers, particularly regarding increased ventricular filling pressures and proximal pulmonary or systemic venous congestion as a result of the PAB and double banding interventions.

### 2.4 Model validation and cross‐species considerations

The simulated pressures used in our study (RV systolic 80 mmHg, LV systolic 180 mmHg) are physiologically relevant and well validated for severe pressure overload conditions. These values align with established precedents in computational modeling studies. Lumens and Delhaas (2012) used similar RV pressure ranges (18–79 mmHg) in PAH simulations with extensive validation against clinical data (Lumens & Delhaas, 2012). Our chosen RV pressure of 80 mmHg represents severe pulmonary hypertension, consistent with clinical definitions of severe PAH (>60 mmHg) (Odeigah et al., 2024; Spyropoulos et al., 2020).

Experimental studies in rat models support the physiological relevance of our chosen pressures. In pulmonary artery banding models, rats develop RV systolic pressures of 60–80 mmHg, representing severe pressure overload (Axelsen et al., 2024; Spyropoulos et al., 2020). Similarly, aortic banding in rats produces LV systolic pressures of 150–200 mmHg (Cho et al., 2014; Kobayashi et al., 1996; Nishimura et al., 2020). These experimental pressure ranges closely match our simulation parameters, validating the cross‐species applicability.

While our study has translational implications for congenital heart disease, we used an adult human model for several reasons: firstly, the fundamental mechanical principles of ventricular interaction are conserved across developmental stages (Arts et al., 2005; Urashima et al., 2008); second, adult models provide a stable baseline for isolating the specific effects of biventricular pressure‐loading; and thirdly, the CircAdapt framework's validated parameters for adult physiology ensure robust computational accuracy. The mechanical insights gained from adult modeling are directly applicable to pediatric populations, and the underlying physiology principles of ventricular interdependence are applicable (Lumens et al., 2019; Munneke et al., 2022).

### 2.5 Animal models

Animal experiments were approved by the St. Michael's Hospital and the Hospital for Sick Children animal ethics committees (ACC942); all experiments were conducted in accordance with each research facility's respective animal research guidelines. All rats were ordered from Charles River Laboratories (strain code: 001). Rats were housed at constant room temperature (21 ± 1°C) with a 12‐h light/dark cycle, and were fed standard chow (Teklad Global 18% Protein Rodent Diets #2018) and water ad libitum. Male Sprague–Dawley rats (312 g ± 21 g; 8–10 weeks old) were randomized to either sham, PAB, or PAB plus TAC double banding (DB) surgical procedures. At the terminal experiment (6 weeks), rats were anesthetized by intubation with a 16G angiocatheter and ventilated with 2% isoflurane and 1.5% oxygen at a rate of 110–120 bpm with a peak respiration pressure of 18–20 cmH_2_O for the duration of the procedure. Sustained‐release buprenorphine (1 mg/kg) and Ketoprofen (5 mg/kg) were administered subcutaneously prior to surgery. Postoperative injections of Ketoprofen were administered every 24 h for 48 h after surgery. Euthanasia was performed by exsanguination (excision of the heart) while animals were under anesthesia following hemodynamic measurements. Male rats were solely used for this study because despite human females predominating in incidence of PAH, males exhibit higher mortality rates, worse RV function, and worse fibrosis, even at mild‐to‐moderate elevations in PVR (Shelburne et al., 2024). This study was conducted in accordance with the ARRIVE (Animal Research: Reporting of In Vivo Experiments) guidelines.

#### 2.5.1 Sham surgery

Animals in the sham surgery group underwent a left thoracotomy. The chest was incised, and a self‐retaining chest retractor was placed. After 5 min of exposure, the chest was closed in two layers using 6‐0 Prolene sutures, and the skin was closed with wound clips.

#### 2.5.2 Pulmonary artery banding

PAB was performed as described in previous work from our group (Sun et al., 2018). In brief, the pulmonary artery (PA) was accessed by left‐sided thoracotomy and dissected free from the aorta. A silk thread was then positioned under the PA with a 16‐gauge needle placed alongside it. A suture was tied tightly around the needle, which was subsequently removed to produce a fixed constriction. The chest cavity was then closed in two layers using 6‐0 Prolene sutures, and the skin was closed with wound clips. Growth of the animal leads to progressive PA constriction over the duration of the experimental protocol.

#### 2.5.3 Double banding surgery

PAB was performed as described above, followed immediately by the TAC procedure. A 7‐0 silk suture was placed around the transverse aorta, between the left carotid and the innominate arteries, and a bent 14G or 12G needle was placed parallel to the aorta. The suture was then firmly tightened around both the needle and aorta, followed by removal of the needle to result in a fixed constriction of the aorta. The chest cavity was then closed in two layers using 6‐0 Prolene sutures, and the skin was closed with wound clips. While the use of two different needle sizes was intended to test different degrees of aortic constriction, we noted that there was no one‐on‐one correlation between end‐systolic pressure (ESP) measured by catheterization (described below) and needle size. For all ex vivo experiments, the surviving animals of the DB group were stratified by RV max pressure/LV ESP (RVP/LVP) ratios, where a larger RVP/LVP indicated a greater degree of RV hypertension and vice versa. Herein, we refer to DB rats that had an RVP/LVP < 0.3 as moderate DB (DBmod), and RVP/LVP > 0.3 as mild DB (DBmild). These ratios were arbitrarily chosen to split the DB group as evenly as possible.

Postoperative analgesia for all groups consisted of subcutaneous injections of slow‐release Buprenorphine at (1.0 mg/kg) and Ketoprofen (5 mg/kg). Additional Ketoprofen (5 mg/kg) was administered subcutaneously for 48 h post‐surgery. Animals from all groups were then maintained for 6 weeks with body weight measurements taken weekly. Animals that survived the 6‐week period underwent echocardiography and hemodynamic testing and were then sacrificed to collect heart and lung tissue, as well as blood samples.

### 2.6 Echocardiography

All surviving animals underwent transthoracic echocardiography 6 weeks post‐surgery. Images were acquired using a high‐frequency ultrasound system (Vevo 2100, MS‐250 transducer, Visualsonics, Toronto, ON). Briefly, echocardiography was performed in rats lightly anesthetized with 1%–1.5 % Isoflurane. The animals were placed in a supine position on a rat physiological monitoring platform equipped with integrated temperature and respiratory sensors, heater, and ECG electrodes (THM150, Indus Instruments, Webster, TX). Body temperature was maintained at 37°C.

Two dimensional long‐axis images of the LV in parasternal short‐axis views with M‐mode measurements at mid‐papillary muscle level were obtained to assess interventricular septum and LV free‐wall thickness, and calculate LV fractional shortening (FS%). Linear dimensions were analyzed offline (Vevo 2100 software v. 1.8). The LV eccentricity index, the ratio of the LV antero‐posterior and septo‐lateral dimensions, was measured during both end‐systole and end‐diastole. Fractional area change (FAC), tricuspid annular plane systolic excursion (TAPSE), and RV free‐wall thickness (FWT) were obtained to evaluate right ventricular (RV) function and hypertrophy.

### 2.7 Hemodynamic measurements

RV and LV hemodynamics were measured 6 weeks following surgery, at no more than 2 days following echocardiography outlined above. Cardiac catheterization was performed as previously described (Connelly et al., 2018). Detailed methods are provided in the Appendix S1.

### 2.8 Histochemical detection of collagen I as a marker of fibrosis

To assess cardiac fibrosis, transverse sections of the hearts (5‐mm thick) were immediately fixed in 10% formalin for 24–48 h and embedded in paraffin wax. Wax blocks were sectioned by microtome (~4 μm per slice). Sections were transferred on glass slides and stained with picrosirius red (PSR) (Polysciences #24901) and imaged (Leica Microsystems A/S, Herlev, Denmark) (Sun et al., 2018). ECM accumulation was quantified in the RV free‐wall, LV, and septal hinge points (HP) using ImageJ (Bankhead et al., 2017).

### 2.9 Protein extraction and immunoblotting

All protein extraction and immunoblotting were performed as previously described (Rzepka et al., 2025).

#### 2.9.1 Antibody list

**Table:** 

Antigen	Company	Catalogue number	Dilution (application)
MRTF‐A	Cell Signalling	14760	1:500 (IB/IF)
YAP/TAZ	Cell Signalling	93622	1:1000 (IB)
GAPDH	Cell Signalling	2118	1:10000 (IB)
pSmad2/3	Cell Signalling	8828	1:1000 (IB)
Smad2/3	Cell Signalling	5678S	1:1000 (IB)
CTGF	abcam	Ab6992	1:1000 (IB)
Anti‐rabbit HRP‐conjugated secondary antibody	Cell Signalling	7074	1:2500 (IB)

### 2.10 Quantitative real‐time PCR (RT‐qPCR)

Total RNA was extracted from rat tissue provided using the miRNeasy Micro Kit (Qiagen, #217084) according to the manufacturer's protocol. Total isolated RNA was reverse transcribed in a 20 μL volume using qScript cDNA Supermix (Quantabio, #101414‐106) according to the manufacturer's protocol. RT‐qPCR was performed with the Applied Biosystems VIIA7 real‐time PCR detection system using Perfecta SYBR Fastmix as a double‐stranded DNA‐specific dye according to the manufacturer's instructions (Quantabio, #101414‐290). In brief, the PCR mixture totalled to 10 μL, containing 2 μL of cDNA or distilled H_2_O (for negative control), 0.25 μL of forward and reverse primers, 5 μL of Perfecta SYBR Fastmix, and 2.5 μL of distilled H2O. mRNA levels were expressed relative to the levels of RPL32. Differences were calculated with the threshold cycle and comparative threshold cycle for relative quantification. All measurements were performed in triplicates. Primer sequences used are outlined in the table below and were supplied by Eurofins Genomics.

#### 2.10.1 RT‐qPCR primer list

**Table:** 

Target	Forward primer	Reverse primer
Rpl32	5′‐TGAAGCCCAAGATCGTCAAAAAG‐3′	5′‐GCACAGTAAGATTTGTTGCACATC‐3′
Col1a1	5′‐TGCCGATGTCGCTATCCA‐3′	5′‐TCTTGCAGTGATAGGTGATGTTCTG‐3′
Col3a1	5′‐CCAACTGGTGGCCAGAATTATT‐3′	5′‐CCATTCCTCCGACTCCAGACT‐3′
Tgfb1	5′‐CTGCTGACCCCCACTGATAC‐3′	5′‐AGCCCTGTATTCCGTCTCCT‐3′
Ctgf	5′‐TAGCTGCCTACCGACTGGAA‐3′	5′‐CTTAGAACAGGCGCTCCACT‐3′
Serca	5′‐GATCCTCATGGACGAGACGC‐3′	5′‐AGGGCTGGAAGATGTGTTGC‐3′

### 2.11 Statistical analysis

GraphPad Prism 9.0 was used for statistical analyses. Data from all experimental groups were subjected to Shapiro–Wilk tests for normality. One‐way ANOVA was used to test for significance using a *p* value of 0.05, and a Tukey multiple comparison test was used for post hoc analysis unless otherwise specified. Outliers were identified using a Grubb's test (*α* = 0.05). All values are expressed as means ± standard deviation unless otherwise specified.

## 3 RESULTS

### 3.1 Pulmonary and double banding simulations

**FIGURE 2:**
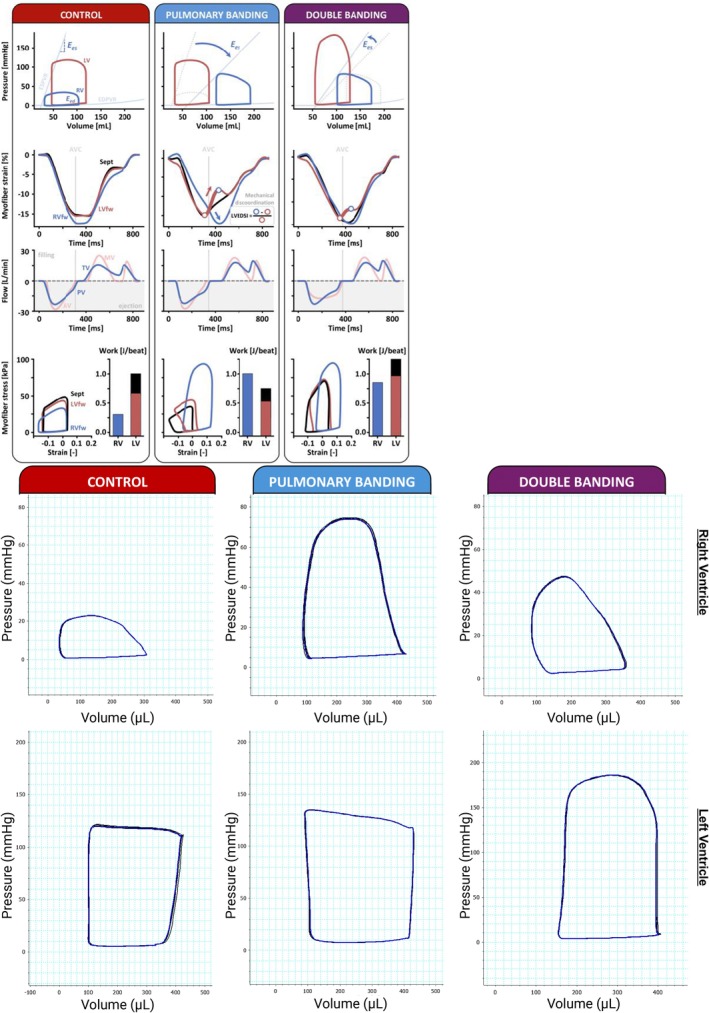
Comparisons between control, PAB, and DB simulations and representative experimental PV loops. Control simulations are depicted in the red column, PAB simulations in the blue column, and DB simulations in the purple column. Rows represent simulated parameters, from top to bottom: PV‐loops of the LV (red) and RV (dark blue), myofiber strain, flow, and myofiber stress. (Middle Column: PAB) Pulmonary banding dilates the RV, reduces RV pump contractility (Ees), induces mechanical discoordination during ventricular relaxation (i.e., increased LVEDSI), increases isovolumic relaxation time and RVfw work density, but decreases LV and septal work density. (Right Column: DB) Double banding reduces RV volume, increases RV Ees, while RVfw myocardial workload (i.e., work density) is decreased. In contrast, the LV dilates and LV myocardial work density increases with aortic banding. The interventricular mechanical imbalance, both in terms of myocardial work density and mechanical relaxation dyssynchrony (LVEDSI) is reduced in the double banding setup, when compared to the pulmonary banding simulation. (Figure 2. Continued) representative RV (top row) and LV (bottom row) PV loops measured from rats that underwent control, PAB, and DB surgeries. AV, aortic valve; DB, double banding; Ees, end‐systolic elastance; LV, left ventricle; LVEDSI, left ventricular end‐diastolic shape index; LVfw, left ventricular free wall; MV, mitral valve; PAB, pulmonary artery banding; PV, pressure‐volume; PV, pulmonary valve; RV, right ventricle; RVfw, right ventricular free wall; Sept, septum; TV, tricuspid valve.

To identify the mechanical underpinnings underlying the impact of DB on the RV, we first used computer modeling (Figure 2) to generate simulated RV and LV pressures, volumes, blood flow across the cardiac valves, myofiber strain, and stress from the CONTROL (left panel), the PAB (middle panel) and the double banding simulations (right panel). In the PAB simulation, the increase in RV afterload resulted in RV dilation and decreased RV pump contractility (E_es_), compared to CONTROL. The strain patterns revealed a more heterogeneous tissue deformation in the three ventricular walls, with RVfw shortening continuing after pulmonary valve closure, prolonged RV isovolumic relaxation, and abnormal early‐diastolic LVfw and septal lengthening (red arrows in Figure 2). The myofiber stress–strain data revealed an increase of peak RV myofiber stress, increased RVfw work, and decreased LVfw and septal work in the PAB simulation compared to the CONTROL simulation.

The addition of LV pressure‐loading to concomitant RV pressure‐loading (Figure 2, right panel) resulted in an acute decrease in RV cavity volume with increased RV contractile function (E_es_), compared to PAB alone. Furthermore, double banding was associated with a less heterogeneous distribution of myocardial tissue load over the ventricular walls, as revealed by a reduced LVEDSI and a more balanced distribution of myocardial work across the RV and LV walls (i.e., reduced RV work in comparison to isolated PAB). However, these improvements in RV function came at the expense of LV dilation and increased LV myocardial myofiber stress and work. Representative RV and LV PV loops from rats that underwent control, PAB, and DB surgeries are included in Figure 2 Continued. Experimental RV loops closely resembled the predicted rightward shifts and vertical elongation predicted by the PAB simulation. In the experimental DB rats, PV loops displayed the predicted leftward shift from the DB simulation. We did, however, observe a lower RV max pressure in the experimental DB rats compared to the simulated DB scenario. Experimental LV loops also resembled the rightward shift and vertical elongation predicted by the DB simulation.

### 3.2 Effect of aortic banding severity on pump function

**FIGURE 3:**
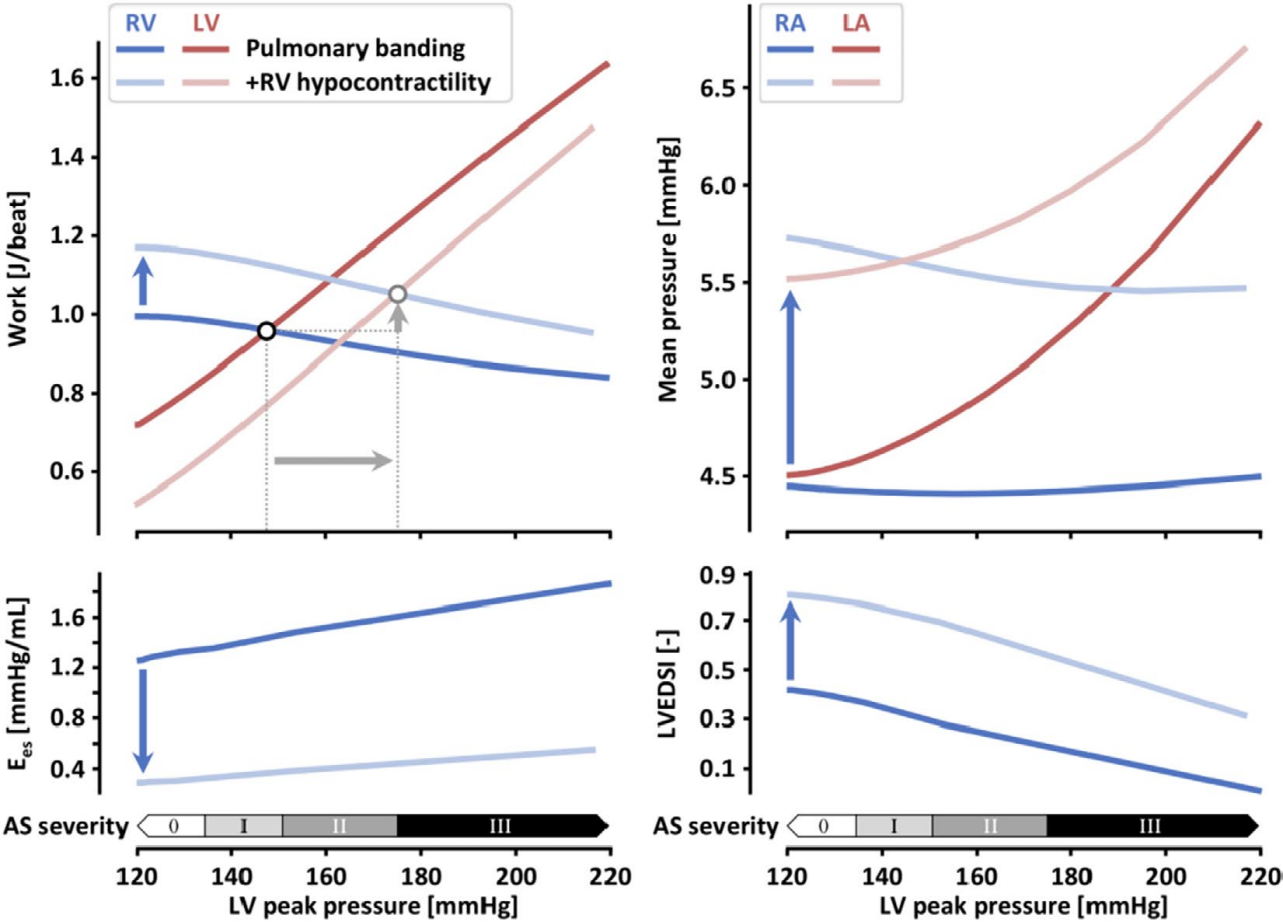
Simulation of varying degrees of aortic banding in combination with PAB with and without RV hypocontractility (solid & light‐shaded lines, respectively). (Left) Increasing aortic stenosis (AS) severity gradually decreased RV work with a greater increase in LV work, resulting in a net increase in total cardiac work regardless of RV hypocontractility. The point at which RV and LV produced equal work was observed at higher aortic banding severity in the presence of RV hypocontractility. (Right) Double banding marginally reduced mean RA pressure with a larger increase in LA pressure regardless of RV hypocontractility. AS, aortic stenosis; LA, left atrium; LV, left ventricle; PAB, pulmonary artery banding; RA, right atrium; RV, right ventricle.

Given the simulation results presented in Figure 2, and toward possible clinical translation of LV pressure‐loading as a treatment for RV dysfunction in conditions of RV pressure‐loading, we further explored the effects of varying degrees of aortic stenosis (AS) combined with PAB. Hence, Figure 3 illustrates the dependence of RV and LV work, mean RA and LA pressures, RV E_es_, and LVEDSI on the degree of aortic banding (stenosis) with concomitant PAB. Furthermore, as RV pressure‐loading conditions are commonly associated with reduced RV contractile function, we simulated the varying degrees of aortic banding in the absence (solid lines) and presence (light‐shaded lines) of intrinsic RV hypercontractility.

Increasing aortic banding severity gradually decreased RV work with a greater increase in LV work, leading to a net increase in total cardiac work, regardless of intrinsic RV hypercontractility. The point of equal work distribution between RV and LV was observed at higher aortic banding severity when intrinsic RV contractile function was impaired. In concordance with Figure 2, the mechanism underlying the work redistribution from RV to LV, underlying the gradual aortic banding severity, follows from improved RV contractile function (E_es_) and reduced ventricular mechanical discoordination (LVEDSI), regardless of intrinsic RV hypocontractility.

Lastly, double banding was observed to slightly reduce mean RA pressure with a larger increase in mean LA pressure, particularly in the presence of intrinsic RV hypocontractility. This suggests that while double banding may alleviate RV workload and congestion, there may be a potential for adverse effects on LV function and hemodynamics.

### 3.3 Descriptive and survivorship data

Given our in‐silico data indicating a mechanical basis for a beneficial impact of DB on RV function, we sought to experimentally validate these findings. A total of 59 rats were operated on for this study. For the sham group (*n* = 5), all rats survived the 6‐week postoperative period. For the PAB group, of the 24 rats that underwent surgery, 14 survived the 6‐week postoperative period and underwent hemodynamic and echocardiographic assessment. For the DB group, of the 30 rats that underwent surgery, 15 survived the 6‐week postoperative period and underwent hemodynamic and echocardiographic assessment. Disparities between the number of surviving animals and final reported *n* numbers were due to technical difficulties in acquiring PV loops and/or echocardiographic images from the rats as well as the elimination of outliers which signified failed stenosis of the PA or aorta.

In the PAB group, five rats died within the 48 h following surgery, with the remaining 5 dying at the 4–5 weeks time‐point. In the DB group, 11 rats died within the 48 h following surgery. At week 4, four rats were euthanized after showing clinical signs of heart failure (tachy‐dyspnea and weight loss). Body weight and age were similar in all surviving animals prior to surgery as well as at 6 weeks post‐surgery. Heart weight/tibia length ratios were significantly greater (*p* < 0.05) in the PAB and DB groups compared to shams Table 1.

**TABLE 1:** Body weight and heart weight/tibia length comparisons between sham, PAB, and DB rats.

	Sham (*n* = 5)	PAB (*n* = 9)	*p* Value^a^	DB (*n* = 6)	*p* Value^b^	*p* Value^c^
Body weight week 0 (g)	296.4 ± 12.8	291.8 ± 12.1	NS	310.9 ± 32.6	NS	NS
Body weight week 6 (g)	483.5 ± 39.1	450.7 ± 41.2	NS	476.6 ± 25.9	NS	NS
Heart weight/tibia length	0.025 ± 0.0023	0.032 ± 0.0038	<0.01**	0.037 ± 0.0036	<0.001***	NS

*Note*: (Top Row, Middle Row) Rat body weights were similar across all experimental groups at week 0 and at the terminal experiment (week 6). (Bottom Row) Heart weight/tibia length ratios were higher in both PAB and DB rats compared to shams (*p* < 0.01 and *p* < 0.001, respectively). Data are presented as mean ± standard deviation. (**p < 0.01; ***p < 0.001 by one‐way ANOVA and Tukey's post hoc test).

Abbreviations: DB, double banding; PAB, pulmonary artery banding.

^a^ PAB versus Sham.

^b^ Sham versus DB.

^c^ PAB versus DB.

### 3.4 Echocardiographic data

Echocardiography was performed at 6 weeks to assess RV and LV morphology and function and are displayed in Table 2. TAPSE was significantly decreased (*p* < 0.001) in PAB rats compared to shams indicating a decline in RV longitudinal systolic function following pressure overload. TAPSE in DB rats was significantly greater (*p* < 0.01) than PAB rats and similar to shams. RV FWT trended upward in the PAB and DB groups and was not significantly different from sham rats. RV end‐systolic area (ESA) increased in PAB rats compared to sham (*p* < 0.0001) and in DB rats compared to sham (*p* < 0.01). RV end‐diastolic area (EDA) also increased in both PAB and DB groups compared to sham rats (*p* < 0.001 and *p* < 0.05, respectively). RV fractional area change (FAC) decreased in PAB rats compared to sham rats (*p* < 0.0001) and for DB rats compared to sham rats (*p* < 0.0001). For LV parameters, cardiac output (CO) was significantly lower in the DB group compared to PAB and shams (*p* < 0.01). Heart rate (HR) was similar in PAB and DB groups and significantly lower in these groups compared to shams (*p* < 0.05, *p* < 0.01, respectively).

**TABLE 2:** Comparison of RV and LV echocardiographic measurements between sham, PAB, and DB rats.

	Sham (*n* = 5)	PAB (*n* = 9)	*p* Value^a^	DB (*n* = 6)	*p* Value^b^	*p* Value^c^
RV echocardiography						
TAPSE (mm)	2.2 ± 0.61	2.0 ± 0.64	0.610	2.3 ± 0.61	0.888	0.035*
RV FWT (mm)	1.1 ± 0.13	1.7 ± 0.5	0.150	1.6 ± 0.58	0.319	0.872
ESA (mm)	13.9 ± 5.7	41.1 ± 7.4	4.58E‐6***	41.6 ± 14.8	1.09E‐3***	0.948
EDA (mm)	31.1 ± 10.8	58.4 ± 9.5	3.43E‐4***	55.5 ± 21.0	0.028*	0.775
FAC (%)	56.2 ± 0.049	29.4 ± 6.5	1.10E‐6***	22.5 ± 12.1	4.76E‐5***	0.274
LV echocardiography						
CO (mL/min)	114.5 ± 4.2	101.2 ± 26.7	0.293	73.0 ± 18.3	0.0013***	0.075
Diameter;d (mm)	8.4 ± 0.56	8.8 ± 0.8	0.353	8.1 ± 0.74	0.332	0.061
Diameter;s (mm)	4.2 ± 0.78	4.9 ± 0.85	0.167	4.7 ± 0.63	0.405	0.463
EF (%)	79.3 ± 6.1	73.6 ± 8.1	0.179	70.3 ± 6.9	0.080	0.699
FS (%)	50.2 ± 6.7	44.8 ± 6.9	0.164	41.5 ± 5.8	0.082	0.574
LV Mass (mg)	1206.1 ± 135.2	1315.4 ± 237.5	0.355	1374.6 ± 210.4	0.470	0.849
LV Mass Corrected (mg)	964.8 ± 108.2	1052.3 ± 190.0	0.355	1099.7 ± 168.3	0.470	0.849
SV (μL)	304.2 ± 28.3	313.1 ± 59.6	0.759	247.8 ± 53.8	0.063	0.044*
V;d (μL)	386.7 ± 56.1	428.3 ± 82.5	0.323	352.3 ± 68.5	0.327	0.056
V;s (μL)	82.5 ± 31.9	115.2 ± 48.0	0.185	104.5 ± 34.5	0.440	0.426
Heart rate (bpm)	378.5 ± 33.2	320.3 ± 53.4	0.041*	294.8 ± 31.4	0.015*	0.738

*Note*: (Top) Echocardiographic assessment of tricuspid annular plane systolic excursion (TAPSE), revealed a rescuing of RV systolic function following DB surgery. (C) RV Free Wall Thickness (FWT) measured from a parasternal long‐axis view identified trending, but insignificant differences between the Sham, PAB, and DB experimental groups. Data are presented as mean ± standard deviation. Grey‐shaded cells are statistically significant (*p* < 0.05 by one‐way ANOVA and Tukey's post hoc test). (*p < 0.05, ***p < 0.001 by one‐way ANOVA and Tukey's post hoc test).

Abbreviations: DB, double banding; EDA, end‐diastolic area; ESA, end‐systolic area; FAC, fractional area change; FWT, free wall thickness; LV, left ventricle; PAB, pulmonary artery banding; RV, right ventricle; TAPSE, tricuspid annular plane systolic excursion.

^a^ PAB versus Sham.

^b^ Sham versus DB.

^**c**^ PAB versus DB.

### 3.5 Hemodynamic data

Invasive parameters of LV and RV hemodynamics and function are depicted in Table 3. RV max pressures were significantly increased in the PAB and DB groups compared to sham (*p* < 0.001 and *p* < 0.01, respectively). Both PAB and DB rats had greater RV EDP compared to shams (*p* < 0.001 and *p* < 0.01, respectively). RV EDP was greater in PAB compared to DB rats (*p* < 0.05). RV dP/dt_max_ significantly increased in the PAB group compared to sham (*p* < 0.05) rats. RV dP/dt_min_ was significantly reduced in both the DB and PAB groups compared to shams (*p* < 0.05 and *p* < 0.01, respectively). RV ESPVR was reduced in DB rats compared to PAB rats (*p* > 0.05) while RV EDPVR was significantly greater in DB rats compared to PAB rats (*p* < 0.05).

**TABLE 3:** Comparison of RV and LV hemodynamics between sham, PAB, and DB rats.

	Sham (*n* = 5)	PAB (*n* = 9)	*p* Value^a^	DB (*n* = 6)	*p* Value^b^	*p* Value^c^
RV hemodynamics						
Max pressure (mmHg)	20.2 ± 1.5	70.0 ± 14.6	6.58e‐4***	53.3 ± 13.1	0.00325**	0.0595
TAPSE/RVSP	0.094 ± 0.024	0.031 ± 0.011	0.0012**	0.051 ± 0.014	0.0885	0.0302*
EDP (mmHg)	1.6 ± 0.32	7.0 ± 2.2	0.0028**	2.1 ± 0.76	0.0016**	0.0140*
Max dP/dt (mmHg/s)	1231.1 ± 213.3	2766.8 ± 937.9	0.0137*	1578.8 ± 269.2	0.0257*	0.0594
Min dP/dt (mmHg/s)	−1007.3 ± 252.0	−2256.3 ± 551.9	0.0024**	−1855.7 ± 427.4	0.0126*	0.234
Tau (ms)	13.3 ± 2.2	18.3 ± 2.2	0.0548	16.2 ± 1.6	0.0831	0.249
ESPVR (linear)	0.06 ± 0.012	0.18 ± 0.057	9.00e‐4***	0.11 ± 0.016	0.00207**	0.0321*
EDPVR (linear)	0.0061 ± 0.0016	0.0087 ± 0.0017	0.0166*	0.012 ± 0.0032	0.0111*	0.0387*
LV hemodynamics						
ESP (mmHg)	102.4 ± 10.9	128.9 ± 8.2	5.46e‐5***	178.2 ± 14.1	3.36e‐4***	3.03e‐4***
EDP (mmHg)	2.6 ± 1.6	11.5 ± 4.6	0.0014**	13.9 ± 3.6	0.0153*	0.361
Max dP/dt (mmHg/s)	6156.9 ± 1003.4	6817.8 ± 848.3	0.181	8395.2 ± 651.0	8.36e‐4***	0.0039**
Min dP/dt (mmHg/s)	−4685.7 ± 947.2	−6086.0 ± 1661.4	0.0903	−8093.2 ± 2371.6	0.0319*	0.0901
Tau (ms)	12.6 ± 2.7	14.9 ± 2.5	0.0948	11.9 ± 2.0	0.3919	0.0382*
ESPVR (linear)	0.3 ± 0.23	0.21 ± 0.11	0.443	0.37 ± 0.22	0.471	0.245
EDPVR (linear)	0.012 ± 0.0049	0.011 ± 0.0058	0.936	0.034 ± 0.013	0.0651	0.0190*

*Note*: (Top) PAB surgery induced a significant increase in both max and min pressures of the RV. DB surgery significantly increased RV pressures relative to sham, however, these changes were less than that of the PAB surgery alone. RV dP/dtMax was significantly increased in PAB animals compared to shams, however, this change was abrogated in the DB rats. EDV was significantly lower in DB rats compared to both sham and PAB animals which coincided with a decrease in stroke volume. (Bottom) LV end‐systolic and end‐diastolic pressures were significantly greater in PAB rats compared to sham. Predictably, LV systolic pressure was greater in DB rats compared to both sham and PAB rats. Tau was significantly decreased in DB rats compared to PAB rats. EDPVR was greater in DB rats compared to both Shams and PAB. Data are presented as mean ± standard deviation. Grey‐shaded cells are statistically significant (*p* < 0.05 by one‐way ANOVA and Tukey's post hoc test). (*p < 0.05, **p < 0.01; ***p < 0.001 by one‐way ANOVA and Tukey's post hoc test).

Abbreviations: DB, double banding; dP/dt_Max_, maximum rate of pressure development; EDPVR, end‐diastolic pressure‐volume relationship; EDV, end‐diastolic volume; LV, left ventricle; PAB, pulmonary artery banding; RV, right ventricle; Tau, time constant of relaxation.

^a^ PAB versus Sham.

^b^ Sham versus DB.

^c^ PAB versus DB.

LV ESP in both PAB and DB groups was higher compared to sham rats (*p* < 0.001). LV ESP was significantly greater in DB rats compared to PAB rats (*p* < 0.01). LV EDP was significantly greater in the PAB and DB groups compared to sham (*p* < 0.01 and *p* < 0.001, respectively) but was not different between each other. LV dP/dt_max_ was significantly elevated in DB rats compared to both sham and PAB rats (*p* < 0.001 and *p* < 0.01, respectively). LV dP/dt_min_ was significantly lower in DB rats compared to both sham and PAB rats (*p* < 0.01 and *p* < 0.05, respectively). LV tau was significantly lower in the DB group compared to the PAB group (*p* < 0.05) and not different between DB and sham rats. LV SV was significantly elevated in the PAB group compared to sham and DB rats (*p* < 0.01 and *p* < 0.05, respectively). LV EDPVR was significantly lower in the PAB group compared to sham (*p* < 0.01) and nearly 3‐fold increased in the DB group compared to both sham and PAB rats (*p* < 0.05 and *p* < 0.01, respectively).

### 3.6 Histology

**FIGURE 4:**
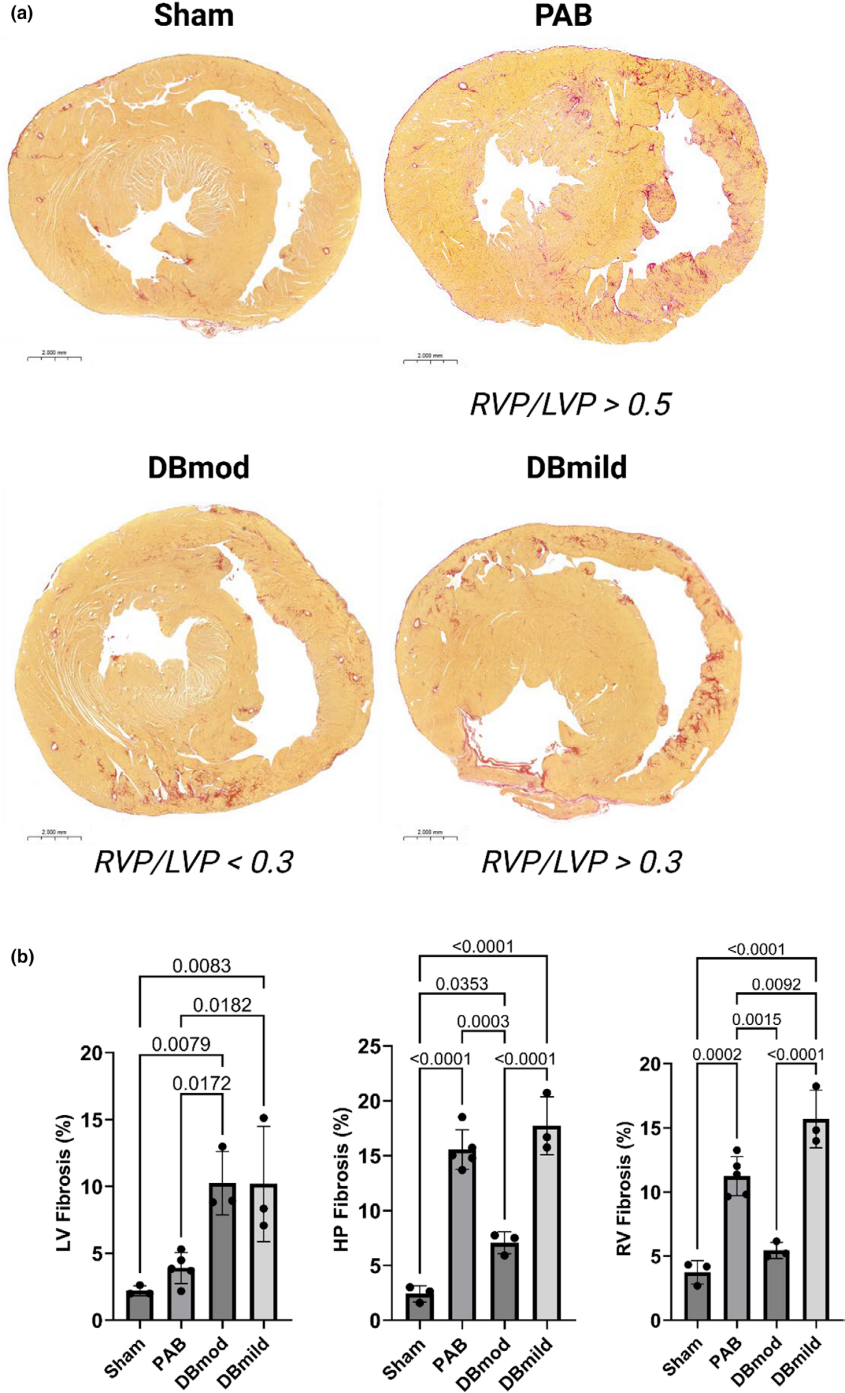
RV fibrosis development is pressure‐dependent. (a) Representative PSR‐stained cross‐sections of rat hearts subjected to Sham, PAB, or DB surgeries. DB group was stratified into moderate (mod) and mild (mild) RVP/LVP ratios to assess the impact of varying degrees of aortic banding concurrent with RV pressure‐loading. (b) Quantification of PSR‐positive stained tissue in the LV, hinge point (HP), and RV free wall. LV fibrosis was highest in the DB groups compared to Sham and PAB. HP and RV free wall PSR‐positive staining was increased in PAB but abrogated in DBmod rats while being higher than PAB rats in DBmild rats. Data are presented as mean ± standard deviation. (*p* values were calculated by one‐way ANOVA and Tukey's post hoc test). Scale bars represent 2000 mm. DB, double banding; HP, hinge point; LV, left ventricle; LVP, left ventricular pressure; mild, mild; mod, moderate; PAB, pulmonary artery banding; PSR, picrosirius red; RV, right ventricle; RVP, right ventricular pressure.

Ventricular cross‐sections stained with PSR are presented in Figure 4. LV collagen content was similar between sham and PAB rats. DBmod rats had significantly lower collagen content compared to PAB within both the RV HP (*p* < 0.001) and RVfw (*p* < 0.01). DBmild rats had greater collagen content in the RVfw compared to both PAB (*p* < 0.01) and DBmod rats (*p* < 0.0001).

### 3.7 Molecular analyses of RV mechanotransduction and fibrosis signaling

**FIGURE 5:**
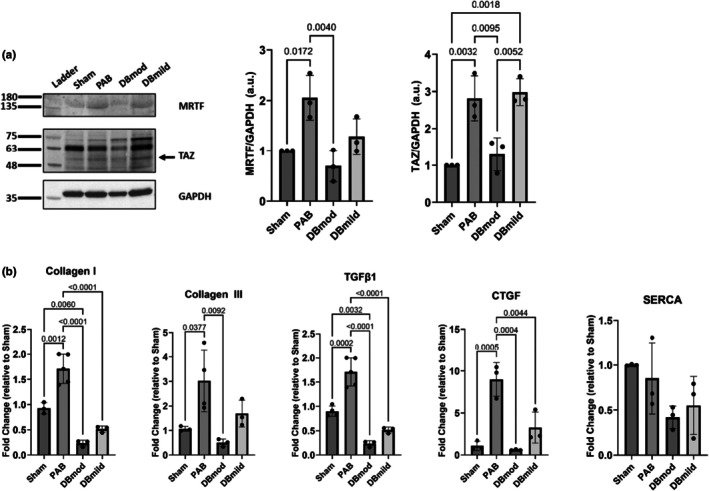
Molecular analyses of Sham, PAB, and DB‐operated rats. (a) Western blotting of MRTF‐A and TAZ, key mechanosensitive proteins of interest to our group, in RV free wall tissue samples revealed a marked increase in MRTF‐A and TAZ levels in PAB rats compared to Shams. This increase was abrogated in DBmod rats. (b) RT‐qPCR of collagens I/III, TGFβ1, CTGF, and SERCA were assessed in RV tissue across all surgery groups revealing clear trends in fibrotic signaling across treatments. DBmod rats demonstrated significantly abolished fibrotic responses in the RV compared to PAB. Data are presented as mean ± standard deviation. (*p* values were calculated by one‐way ANOVA and Tukey's post‐hoc test). CTGF, connective tissue growth factor; DB, double banding; MRTF‐A, myocardin‐related transcription factor A; PAB, pulmonary artery banding; RT‐qPCR, reverse transcription quantitative polymerase chain reaction; RV, right ventricle; SERCA, sarco/endoplasmic reticulum Ca^2+‐^ATPase; TAZ, transcriptional co‐activator with PDZ‐binding motif; TGFβ1, transforming growth factor beta 1.

Western blots of key mechanosensitive transcription factors in RV free‐wall tissue are depicted in Figure 5, top. TAZ protein levels were significantly increased following PAB (*p* < 0.01); this increase was abrogated by the degree of AS, wherein the DBmod group expressed less TAZ protein than the DBmild group (*p* < 0.01) and the PAB group (*p* < 0.01). MRTF‐A followed a similar trend, where DBmod RVs had lower MRTF‐A protein content compared to the PAB group (*p* < 0.01). TAZ protein was not different between the DBmild group and PAB group, indicating a graded effect of LV pressure‐loading on RV mechanosensitive protein expression.

RT‐qPCR data of key pro‐fibrotic genes in RV free‐wall tissue is depicted in Figure 5, bottom. Collagen I was significantly upregulated in PAB rats compared to shams (*p* < 0.01). This increase was abrogated in both the DBmod and DBmild groups (*p* < 0.0001 for both groups vs. PAB). Similar trends were observed in collagen III, TGFβ1, and CTGF. We also evaluated SERCA protein expression given the increase in RV contractility; however, it did not appear to be dependent on RV or LV pressure‐loading.

## 4 DISCUSSION

RV dysfunction and adverse RV‐LV interactions are key drivers of morbidity and mortality in various conditions associated with RV pressure‐loading. While we previously demonstrated the potential to harness RV‐LV interactions for therapeutic benefit in RV dysfunction resulting from RV pressure‐loading, the mechanisms by which this occurs are unknown. Using a combination of computational modeling and empirical experiments, we demonstrated herein that in RV pressure‐loading, the addition of aortic banding results in realignment of the RV and LV mechanical load, with improved RV workload. The improved interventricular and RV mechanics were associated with reduced RV mechanotransduction and profibrotic molecular signaling, with reduced RV collagen deposition and improved RV systolic function.

### 4.1 Effects of aortic banding on the hypertensive RV

The adverse effects of the hypertensive RV on LV have been well described and are important drivers of low LV output and clinical outcomes (Friedberg & Redington, 2014). The dominant adverse RV‐LV interactions stem from a prolongation of RV systole, which, in the presence of RV hypertension, causes a prolonged leftward septal shift. This impairs RV efficient contraction by wasting work on septal displacement. The reduced RV output reduces LV preload, which is worsened by RV peak systole occurring during early LV diastole, critically hindering early LV filling. We recently showed that imbalanced, asymmetrical mechanics within the RV‐between the RV free‐wall and septum, specifically RV myocardial work contributes to RV inefficiency and dysfunction in experimental PAH (Ebata et al., 2021). The computer simulations show that these adverse RV‐LV interactions can be reversed and harnessed by pressure‐loading the LV, which prolongs LV contraction and realigns RV and LV strain deformation. This not only reduces the RV workload but also improves global RV contractility and pump function. Additionally, the beneficial effects of aortic banding on RV function are mediated in part through improved interventricular septal mechanics. Our computational simulations demonstrated that the septum functions as a dynamic load‐sharing mechanism between ventricles. Under isolated RV pressure overload, septal deformation becomes asymmetric with reduced mechanical efficiency, contributing to the observed RV dysfunction (Ebata et al., 2021). However, balanced biventricular loading through aortic banding restores septal work distribution and improves mechanical synchrony, as evidenced by the reduction in LVEDSI in DB versus PAB simulations. Our animal studies partly corroborated these findings, as RV functional improvement was observed with an increased TAPSE compared to PAB alone. The beneficial mechanical effects directly impacted the myocardial response with a reduction in key mechanotransduction and profibrotic signaling, which will be discussed further below. In contrast to the CircAdapt modeling, RV ESPVR decreased in our experimental DB rats compared to PAB rats. This observation may have been due to the DB surgery preventing adverse PAB‐induced remodeling over the 6‐week period or to the lower RV pressures created by the surgery itself. Furthermore, improvements in RV diastolic function were seen in mean RA pressures in the computational modeling, and by RVEDP, and RV EDPVR in the animal experiments. This is clinically important as RV diastolic dysfunction and increased filling pressures are associated with adverse outcomes in PAH. Diastolic dysfunction in RV pressure‐loading results from impaired RV relaxation and reduced RV compliance (Rain et al., 2013; Trip et al., 2015). Indeed, we previously documented an increase in RVEDP in RV pressure‐loading (Akazawa et al., 2020). In the current paper, we confirm that RV‐LV interactions are important not only for LV diastolic dysfunction but also RV diastolic dysfunction. However, the mechanism for improved RV diastolic function in LV pressure‐loading is not via improved relaxation, as in the computer modeling, and animal experiments, Tau was similar in the DB and PAB rats. Rather, the extent of ventricular mechanical discoordination (LVEDSI) is reduced as a direct result of a more balanced and homogeneous tissue load as compared to RV pressure‐loading alone. Thus, the mechanism for improved RV diastolic function and stroke volume in LV pressure‐loading differs from the mechanism of improved RV diastolic function with heart rate reduction, which also improves RV‐LV interactions, but does so via improved RV relaxation; but similar to aortic banding, there is increased RV contractility and reduced RV fibrosis (Gomez et al., 2018; Ishii et al., 2020).

In the current study, the acute increase in PVR led to the expected heterogeneity of tissue load. Previous reports have highlighted RV mechanical dyssynchrony in PAH as a mechanism for RV dysfunction (Adachi et al., 2022; Richter et al., 2020). In the current study, we show that these RV mechanical imbalances extend to hemodynamically and functionally important imbalanced diastolic mechanics between the RV and LV, and provide insights into the potential mechanisms whereby adverse RV‐LV interactions can be addressed and harnessed for therapeutic benefit. Specifically, in RV hypertension, tissue load decreases in the LV and increases in the RV free wall. This leads to RV‐LV mechanical dyssynchrony of myocardial relaxation, indicated by ventricular mechanical discoordination in the myofiber strain plots. Furthermore, consistent with the experimental and clinical observations, an acute increase in RV afterload causes RV dilation and LV compression. By increasing LV pressure‐loading, there is a shift of the RV to lower volumes, with concomitant LV dilatation. Thus, LV pressure‐loading induces mechanical “unloading” of the RV tissue, while RV pressure and CO remained constant. The unloading of the RV tissue by LV pressure‐loading can be seen by (1) decreased workload (area of stress–strain loop = work per unit tissue volume), (2) lower peak stress, (3) less systolic stretch before the onset of myocardial shortening, and (4) less post‐systolic shortening. The improved RV post‐systolic shortening is expected to contribute to improved LV filling in early LV diastole. Previous studies in human patients have shown that the RV afterload is the major determinant of RV wall stress and RV mechanical dyssynchrony in severe PAH (Badagliacca et al., 2015; Richter et al., 2020). In those studies, RV dyssynchrony was weakly related to RV dilatation (Richter et al., 2020), which we now show is also related to imbalanced RV‐LV loading. Our computational findings align with prior studies where RV hypertension causes LV dyssynchrony via delayed early systolic electromechanical activation, late‐systolic septal shift, and postsystolic septal shortening (Frank et al., 2020). These observations are clinically important as PAH patients with greater LV dyssynchrony have worse clinical outcomes and patients with more RV‐LV discoordination have worse exercise capacity (Frank et al., 2020; Liu et al., 2020). While the mechanical effects of the hypertensive RV on RV‐LV diastolic interactions and LV discoordination are dominant, there may be electrical repolarization imbalances that affect diastolic dysfunction (Schäfer et al., 2022). We previously showed that pacing can redistribute heterogeneous and imbalanced RV workload (Lumens et al., 2009; Palau‐Caballero et al., 2017). In the current study, we show that this can also be achieved through modulation of the LV load.

### 4.2 Effects of aortic banding on the LV

The beneficial effects of aortic banding on the RV detailed above come at the cost of LV dilatation, increased LV filling pressures, and LV work. These important adverse effects need to be balanced against the beneficial effects on RV and overall cardiac function. The observed effects in the current study are important as the clinical implication is that LV function needs to be good before increasing its load; otherwise, not only can pressure‐loading further impair LV function, but there will not be beneficial effects on RV function. Nonetheless, our current and previous studies suggested that this can be achieved without the development of LV fibrosis or dysfunction (Apitz, Honjo, Friedberg, et al., 2012; Apitz, Honjo, Humpl, et al., 2012). Adequate LV function is also important in this context as hypothesized additional mechanisms for beneficial LV‐RV interactions invoke shared epicardial myofibers that traverse the ventricles, as well as alleviation of leftward shift (Friedberg, 2018). Thus, the addition of a moderate LV pressure load distributes a part of the extra work from the RV alone to shared compensation between RV and LV. As long as LV function is maintained and the shared compensation does not exhaust the adaptive capacity of the LV, the overall result is beneficial.

### 4.3 Clinical significance

Recently, a number of reports have emerged in the literature regarding PAB in children with end‐stage LV dysfunction (Di Candia et al., 2020; Schranz et al., 2013). While no RCT data is available, there is a clear signal that in appropriately chosen patients, mechanical RV pressure‐loading can lead to improved LV function and clinical outcomes. Such patients include those with PH, as previously discussed, but also patients with congenital heart disease, such as those with Tetralogy of Fallot (TOF), pulmonary valve or branch pulmonary artery stenosis, and transposition of the great arteries (TGA) with pulmonary stenosis or after an atrial switch procedure, all of which are characterized by chronic RV pressure overload. Accordingly, a question arises of whether the principle of loading the contralateral ventricle to preserve function of the diseased one applies to the inverse situation. Our data suggest that there may exist a mechanical mechanism by which this is possible and extends these observations by demonstrating changes in molecular pathways known to be involved in remodeling responses, namely MRTF expression and/or transcriptional activity (Bialik et al., 2019; Rzepka et al., 2025; Speight et al., 2016). Using the CircAdapt model, DB was associated with improvements in myocardial strain, mechanical coordination, and alterations in myofiber stress when compared to PAB alone. These findings are consistent with the current understanding (Palau‐Caballero et al., 2017) of how wall stress activates molecular remodeling pathways, which are initially adaptive, but over time become maladaptive and lead to end‐stage HF and need for transplant or death. The findings are of even greater importance when considering limited evidence based on pharmacologic strategies for preventing end‐stage HF resulting from LV and RV dysfunction. In vivo, our data support physiologic adaptation when aortic banding is added to PAB, with improved RV EDP, TAPSE, and EDPVR. An important observation of our study was that the degree of aortic banding had a differential impact on remodeling responses. This observation aligns with reports of adjustable banding in humans, thus enabling personalized pressure‐loading, which may be optimized to maximize reverse remodeling (Davis, 2008; Talwar et al., 2014). While our findings suggest this mechanistic basis for beneficial RV‐LV interactions, we recognize the inherent limitations in extrapolating from acute in silico and animal models to long‐term clinical outcomes. Systemic hypotension is a well‐established marker of poor prognosis in patients with RV loading, often reflecting advanced disease. Indeed, increasing LV pressure‐loading may be detrimental when LV contractile function is already compromised. Thus, the clinical implications of increasing LV afterload must be interpreted with caution. Our results underscore a need for a nuanced, individualized strategy in optimizing systemic vascular resistance (SVR) relative to specific RV afterload in the patient. The CircAdapt model will be important in clinical management planning and correct patient selection, as it will allow patient‐specific simulations to be performed to optimize hemodynamics prior to invasive or pharmacological procedures being performed.

### 4.4 Molecular responses of DB surgery

Our study provides new insights into how adverse and beneficial RV‐LV interactions impact myocardial remodeling via mechanical transduction. This understanding is important as structural characteristics impact systolic and diastolic function and clinical outcomes. We previously investigated the role of the integrin β1 in triggering RV TGFβ1 and CTGF signaling in the pressure‐loaded RV (Sun et al., 2018). In recent years, research on the role of the mechanosensitive transcriptional cofactors, YAP/TAZ, in the context of heart failure, has revealed complex roles in both cardiac physiology and adverse remodeling, particularly fibrosis (Meng et al., 2022). Studies have indicated that YAP/TAZ activation is regulated by mechanical forces, and inhibition with verteporfin reduces fibrosis in ischemic heart failure (HF) models (Mia et al., 2022). However, this reduction in fibrosis does not always improve cardiac function (Garoffolo et al., 2022). Conversely, fibroblast‐specific knockout of YAP/TAZ reduces fibrosis and preserves cardiac function after myocardial infarction (MI).

The overall result of such roles may depend on genetic ablation, pharmacological inhibition, and in our case, hemodynamic modification. These may be further impacted by differences in animal models (i.e., pressure‐overload vs. ischemia), as well as the multicellular composition of the heart. For instance, the first report of YAP/TAZ involvement in RV responses to PH recently came from Brown et al. (2023). In their study, a calf model of hypoxia‐induced pulmonary hypertension (PH) was used to examine structural and molecular changes in the RV. Using transcriptomics, YAP/TAZ was found to be highly upregulated in the hypertensive RV, and this was coupled to prosurvival, proproliferative, and remodeling responses, suggesting an important role for these signaling molecules in the RV. Our study corroborates this data by demonstrating that TAZ is a mechanosensitive effector within the RV. Additional studies will be required to address the key question of whether modulation of the Hippo signaling pathway in the context of RV PO has beneficial or detrimental impact on RV function and if the overall impact changes with disease development.

Members of our group have previously detailed the mechanisms of action of MRTF‐A/B in rat cardiac fibroblasts and its importance as a mechanosensor (Zhao et al., 2007). Briefly, this study investigated the role of the Rho‐Rho‐kinase pathway in force‐induced smooth muscle alpha‐actin (SMA) expression in fibroblasts (Zhao et al., 2007). The findings revealed that mechanical forces activate the RhoA pathway, leading to LIM kinase and cofilin involvement, ultimately promoting actin filament assembly. This process facilitates nuclear translocation of the transcriptional co‐activator MRTF‐A, activating the SMA promoter and enhancing SMA expression. In continuation, additional work from our group demonstrated that MRTF and TAZ inhibit each other's cytosolic mobility and nuclear accumulation and that TAZ sensitizes Smad3 sensitivity to the SMA promoter, implicating it as a key regulator of myofibroblast character (Bialik et al., 2019; Speight et al., 2016). Our recent work using the rat PAB model in the presence of an MRTF‐A inhibitor demonstrated that RV fibrosis was significantly reduced and accompanied by a mildly improved TAPSE. Furthermore, we identified reduced myofibroblast transition of RV fibroblasts, and that a signaling axis between RhoA, MRTF‐A, and TAZ was central to this effect (Rzepka et al., 2025). These reductions in MRTF‐A and TAZ were observed in our DBmod RV tissue, and to our knowledge, are the first demonstration of MRTF‐A and TAZ upregulation being linked to the balance of pressures between the RV and LV. Additional work would be required to identify other cell types (i.e., cardiomyocytes, endothelial cells, and immune cells) in which these changes in MRTF and TAZ occur, and to further elucidate the ensuing physiological responses by those cells.

### 4.5 Limitations

This work has several limitations. Preclinical studies can capture the complexity of an intervention, and computer models allow understanding of basic pathophysiological mechanisms under well‐controlled conditions. In the computer simulation, we assumed that the LV myocardial tissue was healthy (normal contractility and passive stiffness), whereas previous studies showed that pulmonary hypertension is associated with impaired LV function (Menzel et al., 2000). Second, furthermore, regional electrical and functional differences in ventricular tissue properties were not considered, whereas previous studies have shown delayed average onset of RV activation and regional differences in RV. Third, the computational model assumed a constant heart rate and cardiac output through the use of the pressure‐flow control module, without incorporating autonomic reflexes or cardiovascular adaptation mechanisms such as ventricular remodeling. This design allowed us to isolate the immediate mechanical and hemodynamic consequences of double banding. However, it may not capture longer‐term compensatory responses that occur in vivo. Finally, we did not specifically parameterize the CircAdapt model to represent rodents or pediatric patients. Instead, we deliberately used a validated adult human model to focus on generalizable physiological mechanisms, rather than species‐ or age‐specific differences. While the absolute hemodynamic values may differ across species, the fundamental physical and physiological principles (e.g., Bernoulli valve and Frank‐Starling mechanism) remain conserved (Nishimura et al., 2020). This design choice allowed us to isolate and interpret the key mechanisms of ventricular interaction under pressure overload, without confounding effects of species‐specific adaptations. Future studies could incorporate pediatric‐specific CircAdapt parameters to more directly model congenital heart disease populations.

Importantly, our study only included male rats. Sex differences in PH reveal a complex clinical paradox that significantly impacts disease development, RV function, fibrosis patterns, and mortality outcomes. PAH demonstrates a female predominance, with women being 2–4 times more likely to develop the disease than men (DesJardin et al., 2024; Rodriguez‐Arias & García‐Álvarez, 2021). Despite this increased susceptibility, women with PAH exhibit superior survival rates compared to their male counterparts, creating what researchers term the “sex paradox” or “estrogen paradox” of PAH (DesJardin et al., 2024; Lahm et al., 2014). This paradox extends beyond incidence to encompass RV adaptation, where males demonstrate worse RV function and decreased transplant‐free survival, particularly at mild‐to‐moderate elevations of PVR (Shelburne et al., 2024). The underlying mechanisms appear to involve sex‐specific differences in cardiac fibrosis patterns, with females exhibiting distinct fibrotic responses and better tolerance to cardiac stress (Kostyunina & McLoughlin, 2021). Estrogen‐mediated cardioprotective effects likely explain these survival advantages, as female PAH patients display better RV function, improved hemodynamic reactions to therapy, and reduced RV end‐diastolic pressure compared to males (Kozu et al., 2018). Additionally, sex differences in mortality extend across the PH spectrum, with males consistently showing higher mortality rates even after adjusting for age, disease severity, and treatment factors (Jacobs et al., 2013; Kjellström et al., 2019). We thus emphasize the need for future studies on the RV, especially in a PH context, to include examination of both male and female animals.

Another limitation was that some of the predicted changes in RV physiology from our in silico model were not recapitulated in our experimental model. For example, we experimentally measured an increase in RV ESPVR and EDPVR in the PAB group versus Shams, which were opposite to the CircAdapt simulation. The CircAdapt model simulates acute hemodynamic changes occurring within cardiac cycles, predicting increased ESPVR as an immediate response to pressure overload through the Anrep effect (Yerebakan et al., 2010). In contrast, our 6‐week experimental protocol allowed for chronic adaptive remodeling, during which compensatory mechanisms including concentric hypertrophy, enhanced contractility, and structural adaptations developed. This chronic adaptation can normalize or improve ESPVR compared to acute predictions, as demonstrated in previous studies showing preserved or enhanced RV contractility in compensated pressure overload states (Gaynor et al., 2005; Vonk‐Noordegraaf et al., 2013). The more “normal” ESPVR observed in our DB animals likely reflects successful chronic adaptation rather than acute decompensation, explaining why our experimental results differed from acute computational predictions. Additionally, because we did not include measurements of RV and LV ejection times by echocardiography, we cannot ascertain that the prolongation of RV and LV systole by DB was recapitulated in our in vivo model.

Furthermore, we also recognize the differences in RV pressures created by the PAB and DB surgeries. We hypothesized that this may have been due to survivorship bias wherein rats that achieved higher degrees of PA stenosis died prior to their 6‐week time course. Additionally, other rodent studies that employed aortic banding have carried out postoperative periods of up to 40 weeks (Bosch et al., 2020). As such, the 6‐week timeframe used in our work may have been too short to assess the long‐term impacts of DB on survivorship. Given the highly experimental, technically challenging, and novel nature of the DB surgery, further studies that seek to understand the interplay between pressures in the LV and RV would be well served by assessing the impact of varying needle gauges for both the PA and aortic ligatures.

In conclusion, if LV function is adequate, concomitant moderate LV pressure‐loading may reduce RV dilatation, workload and fibrosis of the pressure loaded RV. The mechanism of these beneficial ventricular‐ventricular interactions seems to be realignment of mechanical events between the RV and LV mechanical load, with improved RV workload, in association with reduced RV molecular mechanotransduction, profibrotic molecular signaling, and collagen deposition along with improved RV contractility.

## AUTHOR CONTRIBUTIONS

Each author has contributed significantly to the successful completion of this study as detailed below: XAL, SR, JFD, TVL, JL, AK, KAC, and MKF contributed to the conception and design of the work. TVL, AP, and JL played a key role in software development for CircAdapt. XAL, SR, JFD, TVL, AP, OK, GK, LN, and JD contributed significantly to the analysis and interpretation of the data. XAL drafted the initial manuscript and incorporated critical revisions. KAC and MKF oversaw the overall project direction and planning, ensuring the integrity of the work. All authors have read and approved the final version of the manuscript.

## FUNDING INFORMATION

XAL, JFD, OK, GK, LN, and KAC were supported by the Canadian Institutes of Health Research (#469998 and #376491). XAL was funded by the CIHR Canada Graduate Scholarship (#457771). SR, TVL, AP, JD, AK, JL, and MKF were supported by the Canadian Institutes of Health Research (#409962). SR, JD, and MKF were supported by the Heart and Stroke Foundation Grant (#G‐24‐0036502).

## CONFLICT OF INTEREST STATEMENT

None of the authors have any conflicts of interest to declare.

## ETHICS STATEMENT

All experimental procedures described in this manuscript were conducted in full compliance with the institutional guidelines for the care and use of laboratory animals. All necessary ethical approvals were obtained prior to the commencement of the study. For animal experiments, efforts were made to minimize animal suffering and to use the minimum number of animals required to achieve meaningful results, in accordance with the principles of the “3Rs” (Replacement, Reduction, Refinement). No conflicting interests that could influence the results or interpretation were identified. All authors have reviewed and approved the final version of this manuscript and agree to be accountable for all aspects of the work.

## Supporting information


Appendix S1.

## Data Availability

The data that support the findings of this study are available upon request from the corresponding author, MKF.
