# Supplementary material for: Left ventricular pressure‐loading improves pressure‐induced right ventricular remodeling by redistributing mechanical load and reducing mechanosignaling
Source: Physiol Rep. 2025 Sep 19;13(18):e70546. doi: 10.14814/phy2.70546 (PMC12447007; doi:10.14814/phy2.70546)
Supplement: Supplementary file 1 — Appendix S1. [file PHY2-13-e70546-s001.docx]

**Supplementary Information**

**Detailed Methods**

***Cardiac catheterization***

Rats were intubated for positive pressure ventilation and placed on a warming pad (42°C). Briefly, rats were anaesthetized with 2% isoflurane and mechanically ventilated (TOPO ventilator, Kent Scientific, Torrington, CT). Rats were kept warm on a heating pad and a 1.4F pressure-volume (PV) catheter (SPR-838; Millar Instruments, Inc., Houston, TX) was inserted into the right carotid, gently advanced into the left ventricle (LV) and PV loops were generated. Data were acquired using a MPVS ultra® PV unit (Millar Instruments) and recorded by a PowerLab 16/35 and LabChart pro (v.8.3) system (ADInstruments Inc., Colorado Springs, CO) under steady-state and during preload reduction. Following a short laparotomy, preload reduction was obtained by compressing the exposed inferior *vena cava* (in between the diaphragm and the liver) using a small cotton swab.

Following LV PV measurements, RV PV measurement was performed using an open-chest method. In brief, the diaphragm was incised using a transverse substernal approach, the pericardium was then removed, and the apex of the RV was punctured with a 26G syringe needle. After removal of the needle, the tip of the catheter was then carefully inserted into the RV and PV loops were generated. Preload reduction was performed by compressing the vena cava using a small cotton swab. The following LV and RV parameters were calculated: end-diastolic and -systolic pressures (EDP, ESP), the maximum rate of pressure rise (+dP/dtmax) and decline (–dP/dtmax), the slope of the end diastolic pressure volume relationship (EDPVR), the slope of preload-recruitable stroke work (PRSW) and Tau (Weiss).

Conductance signals acquired with the Millar catheter were calibrated with the estimated LV volumes derived from echocardiography by using a two-point calibration method, matching LV maximal and minimal conductance signals and end-diastolic and end-systolic volumes (EDV and ESV) measured in long-axis view, respectively. Loops attained from preload reductions were used to obtain end-systolic and -diastolic pressure-volume relationships (ESPVR, EDPVR, respectively).

**Protein extraction and immunoblotting**

All tissues were lysed at a ratio of 50mg of tissue per mL of lysis buffer (20mM HEPES pH 7.4, 150mM NaCl, 1% Triton X-100, 5mM EDTA, 5% Glycerol, supplemented with a Roche complete Mini protease and phosphatase inhibitor cocktail [A32961]). Tissues were lysed by mechanical homogenization followed by sonication on ice. Lysates were centrifuged at 15 000g at 4^o^C for 15min. Protein concentrations in the resulting supernatant were measured by Bradford Assay (Bio-Rad [500001]). Protein samples were mixed with a 4X Laemmli Sample Buffer (62.5mM Tris-HCL pH 6.8, 10% Glycerol, 1% SDS, 0.005% Bromophenol blue, 10mM Dithiothreitol (DTT)), and boiled for 5min at 95^o^C for denaturation.

Protein lysates were loaded into precast Novex 4-20% Tris-Glycine SDS-PAGE gels at 40ug protein per well. Proteins were separated by electrophoresis at 100-130V for 70-90min. Proteins were transferred from onto to a 0.45μm nitrocellulose membrane through semi-dry electrophoresis in a Trans-Blot Turbo Transfer System (Bio-Rad) at 25V for 10min.

Following transfer, membranes were blocked with 5% milk in Tris-buffered Saline + 0.1% Tween-20 (TBST). After blocking, membranes were incubated with primary antibody diluted in blocking buffer overnight at 4oC on a shaker. Primary antibodies used for immunoblotting are described in the table below. The following day, membranes were washed 3 times with TBST, and incubated with secondary antibodies of corresponding species, conjugated to horseradish peroxidase (HRP), diluted to 1:2500 in 5% milk in TBST for 1hr at room temperature. Membranes were washed 3 times with TBST and incubated with Pierce ECL western blotting substrate (Thermo Fisher #32106) for 5 minutes. Membranes were immediately imaged with a Bio-Rad Chemidoc MP using the chemiluminescence setting at optimal autoexposure.

**Full uncut western blot membranes**


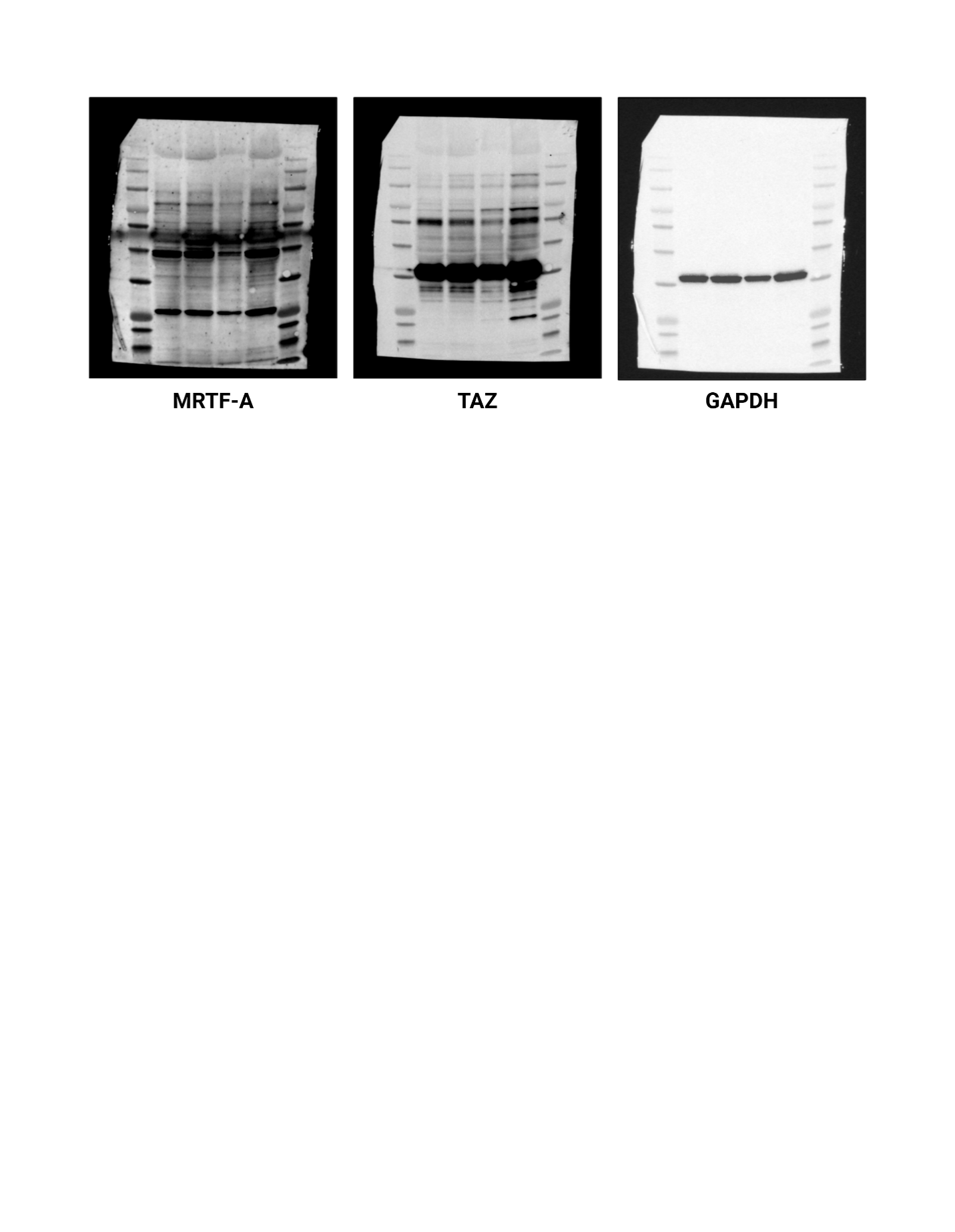


**Figure S1.** Full uncut western blot membranes for MRTF-A, TAZ, and GAPDH in Figure 5.
